# MRI-negative cerebellar syndrome caused by medication-induced magnesium deficiency: a case report

**DOI:** 10.1186/s12883-025-04399-8

**Published:** 2025-09-09

**Authors:** Marvin Jüchtern

**Affiliations:** 1 Department of Neurology, University Hospital, RWTH Aachen University, Pauwelsstrasse 30, Aachen, North Rhine-Westphalia Germany; 2 University Clinic of Neurology, Evangelical Hospital, Carl von Ossietzky University of Oldenburg, Steinweg 13, Oldenburg, Lower Saxony Germany

**Keywords:** Hypomagnesemia, Cerebellar syndrome, MRI, Proton pump inhibitors, Nystagmus

## Abstract

**Background:**

Cerebellar pathologies in adults can have a wide range of hereditary, acquired and sporadic-degenerative causes. Due to the frequency in daily hospital, especially intensive care, settings, electrolyte imbalances are an important, yet rare differential diagnosis. The hypomagnesemia-induced cerebellar syndrome (HiCS) constitutes a relevant disease entity with clinical and morphological variability due to a potential progression of symptoms and a promising causal treatment. Cases of HiCS without imaging abnormalities are scarcely reported and pose a particular challenge to practitioners.

**Case presentation:**

A 68-year-old female patient presented with subacute onset gait impairment and concomitant vertigo. Gaze induced nystagmus, ataxia and limb dysmetria became objectifiable. A broad diagnostic workup, including liquor puncture, whole-body positron emission tomography, antibody serology and most notably thin-layer magnetic resonance imaging remained unconclusive. Only a more detailed examination of chronic hypokalemia with the detection of severe magnesium deficiency under the intake of proton pump inhibitors and a recent gastrointestinal infection found a causal treatment through electrolyte substitution.

**Conclusions:**

Electrolyte disorders as a reason for central nervous pathologies remain underdiagnosed and underestimated, as a heterogeneous clinical appearance, the growing number of defined cerebellar diseases and, like in our case, lacking imaging abnormality aggravatingly contrast with a high intake prevalence of triggering medication. The presence of diarrhea or vomiting, electrolyte shortage, cardiac arrhythmia, alcoholism and particularly the intake of proton pump inhibitors in patients with cerebellar symptoms should result in thorough electrolyte diagnostics. Early recognition of this causally treatable cerebellar syndrome and prompt discontinuation of triggering medication are crucial to improve the often poor prognosis.

## Background

Coordination disturbance, central nystagmus and dysarthric articulation are indicative of a cerebellar pathology with a challenging diversity of possible differential diagnoses. Aside from an (ethyl)toxic, vascular, inflammatory, paraneoplastic and degenerative origin, also late-onset hereditary ataxias, infectious and metabolic causes come into consideration [1, 2]. Increasing knowledge about – often drug-induced – electrolyte imbalances and their neuronal pathophysiology led to a greater awareness of in part rare manifestations of common laboratory aberrations. Although in neurology the focus is mostly on abnormalities of sodium (e.g. central pontine myelinolysis, symptomatic seizures) and potassium (e.g. episodic ataxia, periodic paralysis), magnesium plays a crucial role in ion channels, mitochondria and neuromuscular excitability [3]. In addition, it contributes to endothelial stability and neurotransmitter release, leading to edema and atrophy in magnetic resonance imaging (MRI) in case of deficiency [4]. Beyond typical symptoms as carpopedal spasms, cardiac arrhythmia and bone instability, there are most notably central nervous manifestations of magnesium deficiency, including nystagmus, dizziness, epileptic seizures and confusion. A recent systematic review found that a majority of neurological patients with hypomagnesemia-induced symptoms had movement disorders, among which tremors, dysarthria and ataxia were present in more than a fifth of patients [4]. Furthermore, in intensive care settings, serious magnesium shortage is a relevant complication and associated with increased morbidity and mortality [5]. Magnesium deficiency is not an uncommon laboratory finding in internal and neurological patients, with prevalences ranging from 2.7% in the general population and about 10% in hospitalized patients to up to over half of patients with alcoholism or diabetes [6].

The presented case shall illustrate the possibility of a hypomagnesemia-induced cerebellar syndrome (HiCS) with a rare absence of imaging abnormalities, showing its clinical variability, diagnostic considerations and therapeutic approach.

### Case presentation

A 68-year-old woman presented with an exacerbation of gait instability over the course of nine days. The patient reported a staggering vertigo while sitting, standing and walking and described a lack of coordination of her left leg. She experienced nausea, daily emesis and diarrhea for two weeks before admission. Upon request, she reported a weight loss of 8 kg (17.64 lbs) in the last four months without the presence of fever or night sweats.

Underlying medical conditions included arterial hypertension and gastroesophageal reflux. Alcohol consumption was explicitly denied. Long-term medication comprised antihypertensive, statin and proton pump inhibitor (PPI) therapy.

The clinical examination revealed a horizontal gaze induced nystagmus with a vertical component, atactic pointing and pursuing tests of the upper and lower extremities with a left dominant intention tremor as well as a wide-based atactic gait pattern. The Romberg’s test was insecure with undirected tendency to fall. Vestibular testing remained unremarkable.

There were no abnormalities in cranial computer tomography (CT). Laboratory examinations found moderate hypokalemia (3.4 mmol/l) with normal sodium levels (142 mmol/l). Hypokalemia turned out to be recurring so that a continuous oral substitution was initiated. An investigation of the cerebrospinal fluid (CSF) remained unremarkable.

**Fig. 1:**
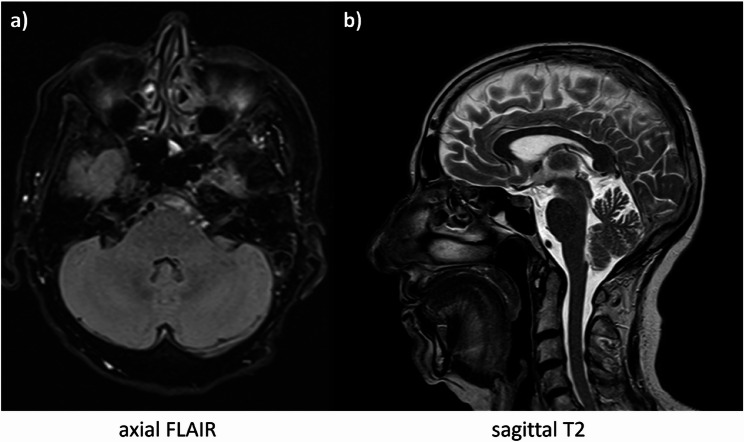
MRI of the patient’s neurocranium. Axial FLAIR- (**a**) and sagittal T2 (**b**) sequences of thin-layered MRI with normal presentation of infratentorial brain structures. Notably, uni- or bilateral hyperintense lesions (usually a sign of vasogenic edema), or signs of cerebellar atrophy were absent despite significant symptoms for more than two weeks. FLAIR = fluid-attenuated inversion recovery.

We conducted a cranial MRI with thin-layered brainstem depiction and four-dimensional angiography that could not find aberrations of infratentorial brain structures even in a short-term follow-up after two weeks. In particular, there were neither signs of vascular, inflammatory or tumorous lesions nor edema. See [Fig. 1] below.

A tumor screening by means of whole-body ^18^fluorodeoxyglucose positron emission tomography (FDG-PET)/-CT, endoscopic examination and paraneoplastic serum antibodies remained negative.

Further laboratory examinations revealed a severe magnesium-deficiency of 0.19 mmol/l (= 4.82 mg/l; reference value 0.66–0.99 mmol/l), in addition to therapy-resistant hypokalemia, which we initially interpreted in the context of a diagnosed Clostridioides difficile infection. Urinalysis solely found hypomagnesuria (< 1.18 mmol/24 h). Through an assessment of previous laboratory findings, we could elicit a chronic magnesium and potassium deficiency due to long-term high-dose daily intake of omeprazole. See Table 1 for details on laboratory values.

**Table 1:** Laboratory findings of the patient over time

Value (Reference value)	On admission	Week 3	On discharge	After 8 months
Sodium (136–145 mmol/l)	142	142	139	142
Potassium (3,5–5,1 mmol/l)	3,5	3,4	3,3	4,7
Phosphate (0,81 − 1,45 mmol/l)	-	1,05	0,79	1,26
Magnesium (0,66 − 0,99 mmol/l)	-	0,19	0,64	0,61
Calcium (total, 2,20 − 2,55 mmol/l)	1,74	1,95	1,85	2,45
Calcium (corrected)	-	2,10	2,08	2,38
Albumin (3,5–5,2 g/dl)	-	3,4	3,1	4,3
Parathormone (15–65 pg/ml)	-	-	143,4	-
25-OH-Vitamine D3 (30–50* ng/ml)	-	-	37,3	-
Creatinine (0,5 − 0,9 mg/dl)	0,58	0,49	0,52	0,62
CRP (< 5,0 mg/l)	94,9	6,4	1,3	3,0

*Reference values are subject to seasonal and laboratory technique fluctuations. This reference follows the recommendations of the Robert-Koch-Institut, Berlin, Germany. Source: https://www.rki.de/SharedDocs/FAQ/Vitamin_D/FAQ07.html (German language)

A therapeutic attempt with intravenous methylprednisolone had been temporarily initiated without satisfactory effects.

After detection of serum magnesium deficiency, we discontinued the PPI and began an intravenous substitution of magnesiumsulfate which was shifted to an oral maintenance treatment upon normalized blood levels of magnesium (0.64 mmol/l). Under this medication there was a rapid amelioration of cerebellar symptoms, with the patient being able to walk medium distances on her own. There was still evidence of a mild intention tremor on the left and a residual horizontal gaze-evoked nystagmus.

In a clinical follow-up visit eight months later, the patient reported only a mild constant gait imbalance; gastrointestinal symptoms, vertigo and nausea ceased permanently. Magnesium substitution and physiotherapy were regularly continued. There were persistent borderline magnesium levels (0.61 mmol/l) due to renewed prescription of omeprazole, which required an increase of the oral magnesium dosage.

## Discussion and conclusions

In synopsis of findings, we diagnosed a subacute hypomagnesemia-induced cerebellar syndrome in our patient. Etiologically we found a long-term high-dose PPI intake, which can be a typical cause of chronic magnesium deficiency [7–11], as well as an acute aggravation resulting from a gastrointestinal infection.

First reports of a cerebellar pathology due to magnesium shortage appeared in the early 1980’s within the context of enteropathies [12] as well as associated with the taking of cyclosporine [13]. Only in the past years, a renewed reception of the phenomenon took place, which was summarized in a review by Kamm and colleagues [7]: In line with our case, HiCS generally presents as a subacute progressive disease with vertigo, nausea, oculomotor deficits (especially downbeat nystagmus [11, 14, 15]) and gait imbalance. The average age of onset was 58 +/- 14 years. In laboratory testing, magnesium levels below 0.2 mmol/l with concomitant potassium shortage were recorded as well as PPI intake as the cause in twelve of 18 cases. For all reported patients, substitution therapy led to a significant improvement of symptoms, albeit – as in our case – nearly half of all patients had residual complaints. In contrast to our patient, there were seven cases who had epileptic seizures, periodic nystagmus was common and notably 14 cases presented with MRI aberrations.

Thus, it’s remarkable that five cases, including ours, had no signs of, mostly bilateral, cerebellar edema, although presenting with significantly disabling symptoms. Described MRI findings, which are typically perceptible in T2- and diffusion-weighted sequences and sometimes recurrent despite treatment, are considered as potentially reversible [7, 8] and had been compared to the posterior reversible encephalopathy syndrome (PRES) [7, 16]. In PRES there is a frequent appearance of T2- and diffusion-weighted (DW-) MRI-hyperintensities without contrast agent uptake, especially in the parieto-occipital cortex, as well as headache, neuropsychiatric impairments, disturbance of consciousness and epileptic seizures [17]. All of these were not seen in the current patient, so we consider HiCS a clinically and etiologically separate disease entity. One hypothesis about the variability of visible brain edema relates to a prominent neuronal hypo- or hyperexcitability as the symptom-determining mechanism without significant endothelial leakage, which was described in a case report of a patient with hypomagnesemia-induced ataxia alongside downbeat nystagmus and myopathy [18], relating to the interaction of magnesium with N-methyl-D-aspartate (NMDA)- and gamma-aminobutyric acid (GABA)-receptors [4]. Moreover, MRI abnormalities can be reversible, sometimes transient throughout the course of disease and might transform into neuronal atrophy [4, 8], so that normal brain imaging should lead to follow-up investigations, since lesions can appear at a later point of time.

Biochemical relations illustrate the (patho)physiological importance of magnesium for the nervous system. While in the periphery it plays a relevant role for neuromuscular excitability which explains symptoms as tetanies, fasciculations and cramps, magnesium-dependent functional disturbances of cell membranes and mitochondria lead to central nervous (CNS) manifestation (e.g., seizures, nystagmus, confusion, choreoathetosis) [3]. HiCS therefore represents a regional CNS variant of hypomagnesemia-related disease, clinically heterogenous and – like in our patient – possibly without an imaging correlate.

Typical causes of magnesium deficiency (lower threshold approx. 0.5–0.66 mmol/l ≈ 12–16 mg/l) are related to gastrointestinal and renal malfunction. Renal causes include hereditary-endocrine diseases (e.g. Gitelman’s syndrome) and most commonly medication (diuretics, cisplatin, aminoglycosides, among others). Gastrointestinal malabsorptive and malnutritive etiologies include the here evinced association with PPI (H^+^/K^+^ ATPase inhibitors), which reduce paracellular magnesium absorption and magnesium-affinity of enterocytes due to a decreased intraluminal pH value [19, 20]. Discontinuation of PPI is an important therapeutical measure to enable adequate absorption of magnesium storage replenishment. Where necessary, especially in refractory cases of reflux disease, dose reduction of PPI and/or administration of lower risk alternatives like antacids or alginates (particularly magnesiumalginate) can be reasonable options [21]. Other causes of hypomagnesemia can be hyper(para)thyroidism, albumin deficiency and type 1 or 2 diabetes [3].

Aside from treatment of triggering factors, hypomagnesemia therapy primarily builds upon substitution. Especially in cases of severe and rapidly evolving shortage and/or malabsorptive causes, an initial treatment with intravenous magnesium, e.g., 2 g magnesiumsulfate and thereupon 4–6 g (depending on kidney function) per day for three to five days [3] appears appropriate. It should be followed by an oral long-term therapy, e.g., with 250-400 mg two times a day or weight-adapted (e.g., 4-6 mg per kilogram body weight per day [22]), for which different magnesium salts are available. Due to the potentially higher bioavailability, organic bound magnesium salts (e.g., magnesiumcitrate, -gluconate, -orotate) have been preferred by some [23]. Side effects of substitution can be nausea, vomiting and diarrhea; intoxication is possible and presents with hypotonia, muscle weakness, cardiac arrhythmia and – in severe cases – disturbance of consciousness [22].

In conclusion, hypomagnesemia represents an underestimated, yet clinically relevant cause of cerebellar symptoms, even if no imaging aberrations can be found. Thus, the following anamnestic, laboratory and clinical findings should remind of HiCS: A subacute cerebellar syndrome with or without epileptic seizures, diarrhea/emesis, cardiac arrhythmia, hypokalemia, intake of proton pump inhibitors or diuretics, diets and gastrointestinal malabsorption and alcoholism. A diagnostic workup should be parallelly conducted to a standard, syndrome-specific investigation and should comprise electrolyte analysis, thyroid and parathormone diagnostics, diabetes parameters, carbohydrate-deficient transferrin (CDT), vitamin levels, liver values and urinalysis. A follow-up of FLAIR and DW-MRI should be considered since morphological signs of HiCS can be transient. Treating physicians ought to discontinue potentially causal medication and initiate magnesium substitution as early as possible to avoid frequent symptomatic progression.

## Data Availability

MRI data generated during this study are included in the published article. All datasets used during the current study are available from the corresponding author on reasonable request.

## Abbreviations

MRI: Magnetic resonance imaging
HiCS: Hypomagnesemia–induced cerebellar syndrome
PPI: Proton pump inhibitor
CT: Computer tomography
CRP: C–reactive protein
CSF: Cerebrospinal fluid
FDG: PET–^18^Fluorodeoxyglucose positron emission tomography
FLAIR: Fluid–attenuated inversion recovery
PRES: Posterior reversible encephalopathy syndrome
DW: Diffusion–weighted (MRI)
NMDA: N–methyl–D–aspartate
GABA: Gamma–aminobutyric acid
CNS: Central nervous system
CDT: Carbohydrate–deficient transferrin
